# Editors’ selection of papers from China's academic journals

**DOI:** 10.1093/nsr/nwz027

**Published:** 2019-03-15

**Authors:** Yuan Gao

**Affiliations:** Editors’ selection of papers from China's academic journals

## PHYSICS

### 2D gallium oxide memristors for neuromorphic computing

**Figure 1.**
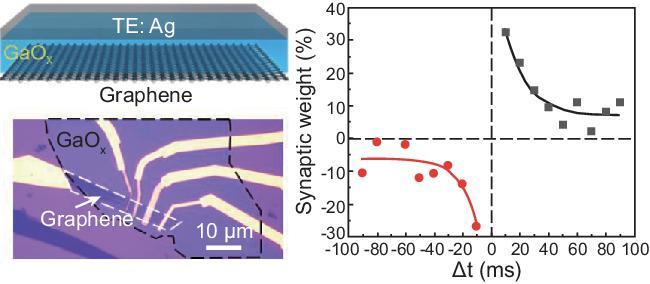
The optical image and schematic diagram of the graphene/GaOx/Ag memristor. The STDP synaptic function is realized in the memristor device.

Synapse emulation is very important for realizing neuromorphic computing, which could overcome the energy and throughput limitations of today's computing architectures. Recently, a research team led by Guangyu Zhang at the Institute of Physics, Chinese Academy of Sciences (CAS), reported the realization of the synaptic function in memristor based on a vertical structure of graphene/ultrathin-gallium-oxide/Ag (see Fig. 1). The ultra-thin gallium oxide film is produced by a squeegee approach. The synaptic weight of the memristor could be tuned by the applied voltage pulse, number, width and frequency. Such ultra-thin synaptic devices provide a promising platform for developing neuromorphic computers with low energy consumption and high-efficiency.

[Wang S *et al. Chin Phys B* 2019; **28**: 017304]

## PHYSICS

### A large rare-earth-based family of quantum spin liquid

Quantum spin liquid (QSL), theoretically proposed over 40 years ago, is a novel spin-entangled phase exhibiting exotic excitations. Searching for ideal QSL materials is still a major challenge of the field. Recently, Qingming Zhang (Institute of Physics, CAS; Lanzhou University), Gang Chen (Fudan University), Hechang Lei (Renmin University of China), Xiaoqun Wang (Shanghai Jiao Tong University) and co-workers revealed a large family of triangular lattice QSL materials named rare-earth chalcogenides. The high symmetry, the simplest structure and the rich diversity enable the family to provide a promising QSL platform beyond the existing ones for exploring fundamental issues on QSL relevant to high-temperature superconductivity and topological quantum computing.

[Liu W *et al. Chin Phys Lett* 2018; **35**: 117501]

## CHEMISTRY

### Synthesis of chiral diarylmethylamines by direct asymmetric amination of racemic diarylmethanols

Direct asymmetric amination of racemic secondary alcohols represents a highly expedient strategy for the synthesis of chiral amines—a family of important molecules with strong relevance to human health. However, substantial challenges must be overcome when applying this strategy in the synthesis of chiral diarylmethylamines. Recently, Jianwei Sun and co-workers from the Hong Kong University of Science and Technology successfully achieved this transformation with both high chemical efficiency and excellent enantioselectivity by organocatalysis. A formal asymmetric S_N_1 mechanism involving quinone methide intermediate was regarded as the key to success.

[Chen M *et al. Chin J Chem* 2018; **36**: 587–93]

## CHEMISTRY

### C(sp^2^)−H phosphorylation for direct construction of aminophosphonic acid precursors

Construction of organophosphorus compounds is of great significance in pharmaceuticals, agrochemicals and material sciences. However, the efficient phosphorylation of alkenyl C(sp^2^)−H bonds remains a huge challenge. Recently, Jun-An Ma, Fa-Guang Zhang and co-workers from Tianjin University reported a Pd-catalysed direct and efficient phosphorylation of enamido C(sp^2^)–H bonds to access a considerably wide range of β-amidovinylphosphonates without the need for additional ligands or directing groups. The novel method provides a practical, general and applicable route to the formation of C–P bonds and may find wide applications in relevant fields.

[Qiao B *et al. Chin J Chem* 2018; **36**: 809–14]

## AGRICULTURAL SCIENCES

### Evolution of drought resistance in rice: from theoretical research to breeding practice

**Figure 2.**
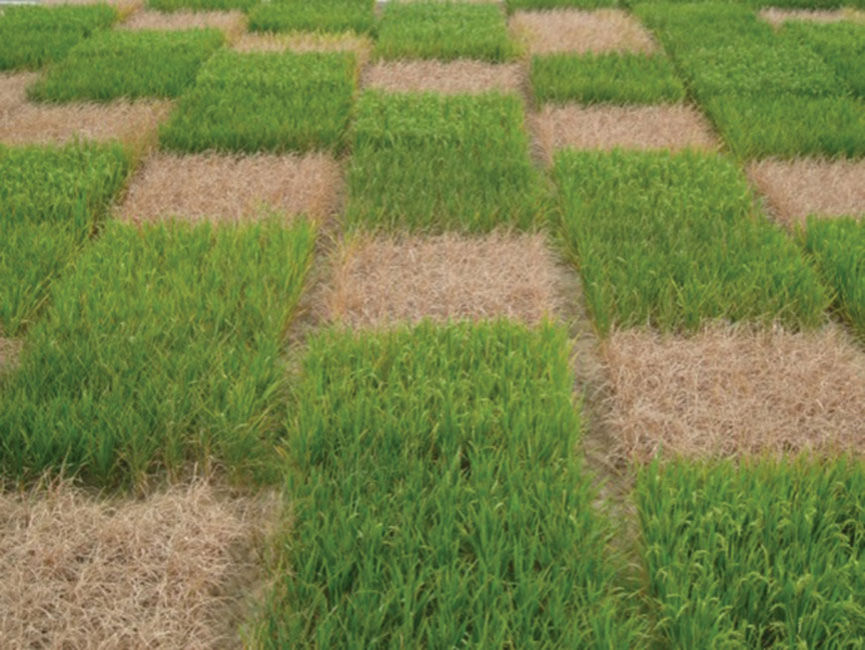
Field performances of water-saving and drought-resistant rice and elite paddy rice under severe drought stress.

It is essential to learn the evolutionary process of drought resistance in rice for breeding drought-resistant rice. The recently published work by Lijun Luo's group from Shanghai Agrobiological Gene Center discloses the mode of bi-directional selection in upland rice for both drought resistance and productivity, which makes it adaptively differentiated from lowland rice in drought resistance (see Fig. 2). The bi-directional selection retains great genetic biodiversity of drought resistance in upland rice. Some upland-specific recombination events can overcome the tradeoff between drought resistance and productivity. By applying the bi-directional selection, the drought-resistant and water-saving rice derived from upland × lowland rice obtains advantages in both drought resistance and productivity.

[Xia H *et al. Mol Plant* 2019; **12**: 170–84]

## NEUROSCIENCE

### Revealing molecular landscapes of cerebellar development through single-cell transcriptome analysis

The cerebellum is critical for controlling motor and non-motor functions via a cerebellar circuit that is composed of defined cell types, which approximately account for more than half of neurons in mammals, whereas the molecular mechanisms governing cerebellar neuronal fate determination and maturation remain not completely understood. Recently, a study led by Zhen-Ge Luo at the Institute of Neuroscience, CAS, and ShanghaiTech University, analysed transcriptome profiles of 21 119 single cells of the postnatal mouse cerebellum and revealed trajectory hierarchies of various cerebellar cell types. The datasets implied roles of mitochondrion and ATPases in the maturation of Purkinje cells (PCs), the sole output cells of the cerebellar cortex, and found enriched expression of ataxia-related genes in PCs. This study will expedite the understanding of mechanisms of cerebellum development and accelerate the understanding of molecular and cellular mechanisms of cerebellar diseases.

[Peng J *et al. J Mol Cell Biol*; doi: 10.1093/jmcb/mjy089]

## GEOSCIENCES

### Global ocean warming broke the record in 2018

The abundant greenhouse gasses emitted by human activities trap more heat in the Earth, driving climate change. More than 90% of the extra heat is stored in the ocean, so ocean heat content (OHC) change is a fundamental climate indicator. An international group led by the Institute of Atmospheric Physics, CAS, released the 2018 OHC data showing that the year 2018 was the hottest year ever recorded for the global ocean. Increases in ocean heat are incontrovertible proof that the Earth is warming and there are real consequences. Actions are needed to minimize future warming trends.

[Cheng LJ *et al. Adv Atmos Sci* 2019; **36**: 249–32]

## MATERIALS SCIENCE

### An effective molecular design strategy to significantly improve photovoltaic performance of a twisted polymer

In polymer solar cells (PSCs), the conjugated polymers with highly twisted backbones are facing a big challenge to realize high power-conversion efficiencies (PCEs), due to their low charge-carrier mobilities and poor photoactive layer morphologies. Recently, Jianhui Hou (Institute of Chemistry, CAS), Wei Ma (Xi’an Jiaotong University) and co-workders successfully improved twisted polymer charge-carrier mobilities and photoactive layer morphologies by introducing C=O units as conjugated side chains to enhance the intermolecular interactions. PCEs of the twisted polymer were dramatically increased from 1.05 to 11.77%. This work shows that the twisted conjugated polymers could serve as promising candidates for high-efficiency PSCs.

[An C *et al*. *Sci China Chem* 2019; **62**: 87–94]

## MATERIALS SCIENCE

### A nanoreactor of hierarchically hollow spheres as metal-free oxygen-reduction electrocatalysts

**Figure 3.**
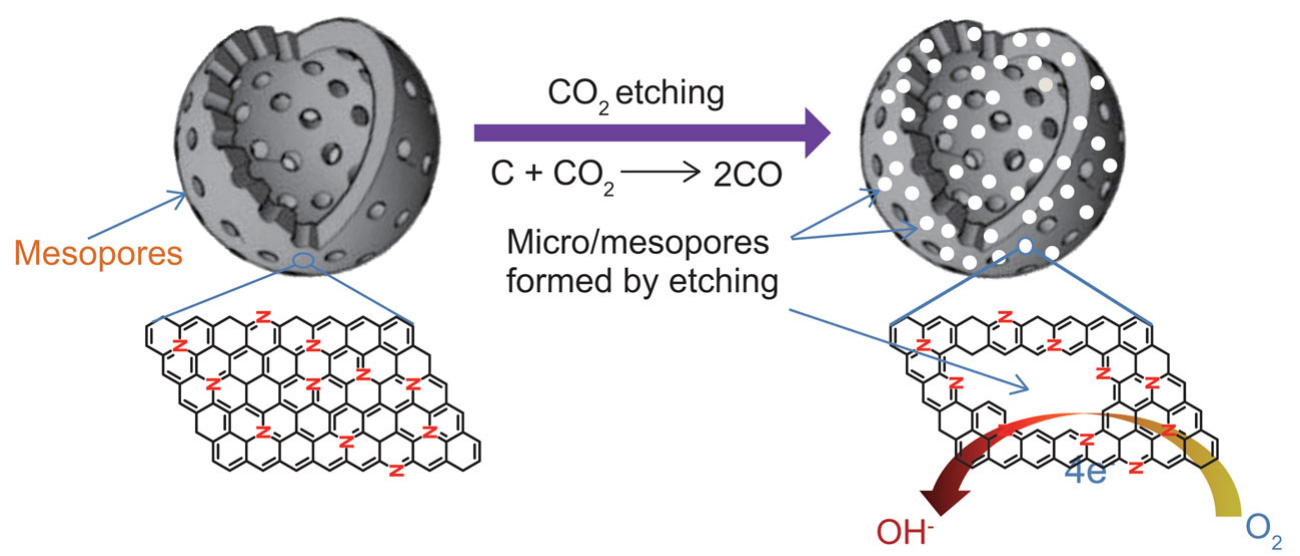
Etching mesoporous shells by CO_2_ to form triple hierarchical electrocatalysts with hollow macroporous cores and hierarchical micro-mesoporous shells, showing improved activity for the ORR via a direct 4^−^-electron reaction pathway.

The creation of hierarchical structures in cathodic electrocatalysts could provide increased density of active sites and faster mass transfer, thus greatly improving the reactivity. Recently, Jiacheng Wang and Qian Liu at Shanghai Institute of Ceramics, CAS, prepared N-doped hollow carbon spheres via CO_2_ activation, showing unique triple hierarchical micro-meso-macroporous structures and large surface areas (see Fig. 3). The optimized catalyst demonstrated comparable oxygen-reduction reaction (ORR) activity but superior methanol tolerance and long-term durability to commercial Pt/C with a 4e^−^-dominant transfer pathway, indicating its great potential as a cathodic electrocatalyst in fuel-cell applications.

[Xing R *et al*. *Nano-Micro Lett* 2018; **10**: 3]

## MATERIALS SCIENCE

### Sb_2_S_3_ hollow microspheres as anode materials for lithium/sodium-ion batteries

The design and synthesis of electrode materials with 3D hierarchical architectures have attracted considerable attention. Recently, Li Liu and co-workers at Xiangtan University fabricated Sb_2_S_3_ hollow microspheres by a simple hydrothermal reaction using SbCl_3_ and Lcysteine as raw materials without adding any surfactants. The novel architecture combines the merits of nanometer size, hollow interior and 3D hierarchical structure. The material presents remarkable cycling performance and outstanding rate capability in lithium-ion batteries and also exhibits superior sodium-storage capabilities in sodium-ion batteries.

[Xie J *et al*. *Nano-Micro Lett* 2018; **10**: 12]

## INFORMATION SCIENCE

### Deep learning helps to discover the creation era of Dunhuang murals

**Figure 4.**
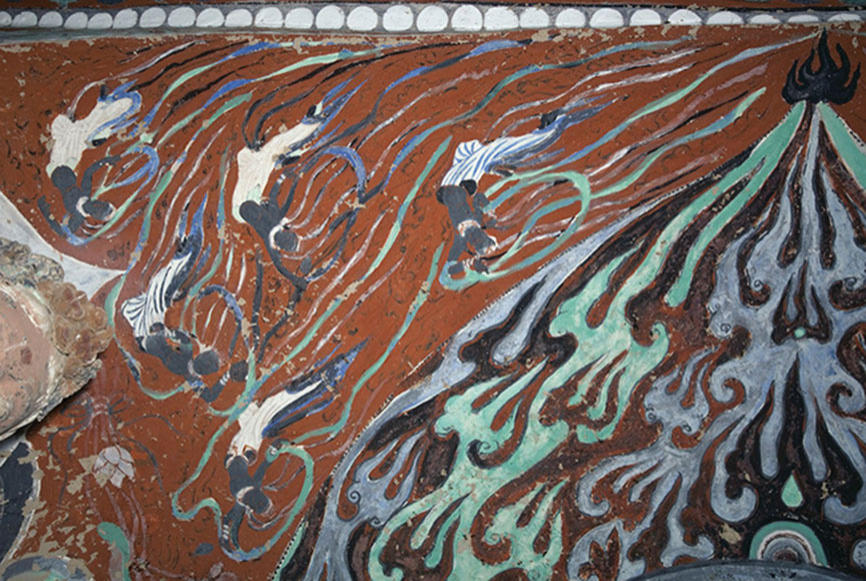
Sui or Tang Dynasty? A mural of Flying Apsaras at Mogao grotto #206 with controversy in its creation era.

Dating the creation era of ancient paintings is very important in archaeology. Some murals at Dunhuang, China, are hard to date due to the lack of reference materials. Recently, Qingquan Li (Shenzhen University), Qin Zou (Wuhan University) and co-workers formulated the problem of mural-painting dating into a problem of drawing-style classification and developed a novel dating method that encodes drawing styles with visual codes learned through deep learning. This new method successfully uncovered the creation era of six mural paintings at Mogao Grottoes (see Fig. 4).

[Li Q *et al*. *Sci China Inf Sci* 2018; **61**: 092105]

